# Treatment-related changes towards normalization of the abnormal external signal processing in panic disorder

**DOI:** 10.1371/journal.pone.0227673

**Published:** 2020-01-27

**Authors:** Christian Valt, Dorothea Huber, Birgit Stürmer

**Affiliations:** International Psychoanalytic University Berlin, Berlin, Germany; Medical University of Vienna, AUSTRIA

## Abstract

Despite the scientific consensus on the efficacy of psychotherapy for the treatment of psychological disorders, the evidence of treatment-related changes towards normalization of abnormal brain functions in patients is mixed. In the present experiment, we investigated whether treatment can affect early information processing, by testing abnormal event-related potentials (ERPs) evoked by internal and external signals in panic disorder. Sixteen patients with panic disorder and comorbid personality disorder and sixteen control participants performed a response-choice task and a passive viewing task in two testing sessions, separated by around 14 months. During this period, patients received psychological treatment. In agreement with previous studies of performance monitoring, the abnormal amplitude of the Ne/ERN–an index of error processing based on internal signals—did not change between the first and second testing session. However, treatment-related changes were evident for the abnormal vertex positive potential (VPP) evoked by external signals in the response-choice task and the passive viewing task. In patients, the VPP was smaller in the second session compared to the first session, whereas no significant changes occurred in controls. This result supplies evidence of treatment-related changes towards normalization in the early information processing of external visual stimuli in panic disorder.

## Introduction

Psychotherapy is a conventional treatment of many psychological disorders. However, it is still unclear whether and how the positive effects of psychological interventions on the well-being of patients correspond to significant changes in brain functioning. Many neuroimaging studies have reported evidence in support of the idea that treatment could lead to a normalization of brain activities, but the evidence of significant changes in early information processing is lacking. In the present study, we employed electroencephalography (EEG) to investigate the effect of treatment on the abnormal electrophysiological response evoked in panic disorder by the processing of internal and external signals related to performance monitoring and by the processing of visual material for passive viewing.

The benefit of psychological treatments on brain functioning is a topic of recent research interest. Neuroimaging studies have shown that psychotherapy might induce changes towards normalization of abnormal brain responses [for a review, see 1]. For example, studies with patients suffering from a major depressive disorder have shown that psychotherapy leads to the normalization of the activity of the amygdala in processing emotional material [2, 3]. These neuroimaging observations of treatment-dependent changes of abnormal brain activity have found further support in a recent electrophysiological investigation. Buchheim and collaborators [4] reported that a 15-weeks-long psychodynamic therapy determined a normalization of the abnormal amplitude of the late positive potential, an event-related potential (ERP) associated with the late evaluation of emotional stimuli [5]. Moreover, Buchheim and collaborators [4] observed changes in the gamma band of the EEG—an index of disengagement from rumination of emotion-related material [6]. Therefore, in major depressive disorder, a psychological intervention seems to be effective in normalizing the brain activity evoked by emotional stimuli. However, despite this recent study, the evidence of further significant changes of abnormal electrophysiological responses is still insufficient, particularly regarding the early processing of internal and external stimuli. For example, in performance monitoring, no study has yet found any evidence of significant changes of the enhanced ERPs evoked by the analysis of internal signals in patients suffering from an internalizing psychological disorder [7–11].

Performance monitoring is a research field that is attracting growing interest because of the close link between abnormal processing of errors and internalizing psychopathologies [12, 13], with promising implications for the early diagnosis of psychological fragilities [14, 15]. A reliable finding in studies of performance monitoring in internalizing disorders is an enlarged electrophysiological response evoked by the processing of internal signals, particularly in the occurrence of errors [12, 13, 16]. Compared to control participants, patients with anxiety or depressive symptoms present a more negative error-related negativity (Ne/ERN), an ERP evoked at around 50 ms after incorrect responses over medial fronto-central recording positions [17, 18]. According to the reinforcement-learning theory, the Ne/ERN reflects a dopaminergic signal, sent from subcortical to cortical brain regions, which calls for adjustments of inaccurate performances [19]. Starting from the evidence of abnormal processing of errors in internalizing psychopathologies, some recent studies have investigated whether short periods of treatment induce a normalization of the augmented amplitude of the Ne/ERN in patients [7–11]. Despite substantial reductions of the symptoms after psychotherapy, these studies reported no significant effect of treatment on the Ne/ERN amplitude. Based on the observed robustness of the Ne/ERN to the remission of symptoms, researchers concluded that this ERP is an endophenotype of internalization disorders [20]. However, by focusing on the processing of errors based on internal signals, these studies did not investigate whether treatment produces functional changes in other abnormal brain responses.

In many circumstances, the brain does not monitor performance exclusively based on internal signals; external signals, like feedback or outcomes, are valuable additional sources for performance monitoring. The investigation of external signal processing in psychopathologies characterized by internalization has shown that also the processing of external signals is abnormal in patients [12, 21]. For example, some studies showed that the amplitude of the feedback-related negativity [FRN, 22]—an ERP that seems to be the feedback-locked counterpart of the Ne/ERN [23, 24]—is significantly larger in patients than in controls, but other studies observed reduced or normal FRN amplitudes [12].

Recently, we investigated the processing of internal and external signals in patients who had panic disorder with comorbid personality disorder before the beginning of psychotherapy [25]. The study aimed to investigate whether patients had a deficit in the flexible processing of internal and external signals and whether potential abnormal processing of external signals was restricted to the context of performance monitoring or extended to passive viewing as well. To this end, we designed an experiment with a response-choice task with informative and uninformative feedback and a passive viewing task with pictures of faces or houses. In the response-choice task, participants had to react to the identity of a target letter, and they received feedback after each response. Here, we tested the flexible allocation of attentional resources by directing the participants’ monitoring focus from internal signals, when feedback was uninformative, to external signals, when feedback was informative. In the passive viewing task, we investigated whether the potential abnormal processing of external signals also occurred for stimuli without any meaning for performance monitoring or any biological relevance, such as pictures of houses. In agreement with previous studies of error processing in internalization psychopathologies, compared to control participants, patients presented a larger Ne/ERN evoked by the monitoring of errors based on internal signals. However, despite the lack of any difference in the FRN amplitude, the vertex-positive potential [VPP; 26] evoked by informative and uninformative feedback was more positive in patients compared to controls. The VPP is an ERP elicited over medial fronto-central recording positions at around 200 ms after the onset of a visual stimulus, and it reflects the allocation of attention for early information processing [27]. Importantly, the VPP is thought to be the fronto-central counterpart of the N170 [28]; in fact, they are generally reported as the N170/VPP complex [29]. In the context of face processing, the N170 and the VPP are thought to reflect an early perceptual stage of visual processing that feeds the subsequent cognitive evaluation of the stimulus, for example, in the interpretation of the affective valence of emotions [30]. Therefore, the augmented Ne/ERN elicited by incorrect responses and the enhanced VPP evoked by visual stimuli in patients suggest that enlarged processing of internal signals associated with errors and heightened vigilance to external stimuli are two characteristic features of panic disorder. Besides the significant differences between groups in the Ne/ERN and the VPP amplitudes, patients and controls showed comparable ERP modulations according to the feedback information content, suggesting a normal capacity to take into account contextual factors for the flexible processing of internal and external signals. Interestingly, in the passive viewing task, patients also presented an enhanced VPP evoked by processing pictures of houses and faces. This result suggested that the deficit in patients was not restricted to the context of feedback processing because it also occurred during the passive viewing task and that it did not only affect the processing of faces because it applied to pictures of houses as well.

The present experiment aimed to investigate the potential effects of treatment on the analysis of errors based on internal signals and the processing of visual material, such as feedback in the response-choice task or pictures in the passive viewing task. Participants (patients and controls) who took part in the study conducted by Valt and collaborators [25] were invited for a second testing session of the same experiment circa one year after the first testing session. Importantly, during the interval between the first and second testing sessions, patients received psychotherapy, with pharmacotherapy when indicated. The experimental designs in the two testing sessions were identical, but, in this study, we focused on the abnormal amplitudes of the Ne/ERN and the VPP to detect potential treatment-related changes in patients. On the one hand, in agreement with previous studies on the perception of external stimuli [2–4], we expected to find significant normalization of the augmented VPP in patients in both the response-choice task and the passive viewing task, as an indication of a treatment-related change of early information processing of visual material. On the other hand, according to previous ERP studies on the monitoring of errors, we did not expect any effect of treatment on the abnormal amplitude of the Ne/ERN [7–11]. The results of the present experiment should clarify whether, compared to the insensitivity of the Ne/ERN to treatment, the VPP represents a more suitable electrophysiological marker for studying treatment-induced changes of abnormal brain functioning in patients.

## Materials and methods

### Participants

The twenty-two patients that took part in the study before the beginning of therapy [25] were invited for a second testing session approximately one year after the first experiment. Sixteen patients accepted the invitation and attended the second testing session. Fifteen out of the sixteen controls agreed to participate in the second testing sessions, but one control participant did not reply to the invitation. As a replacement, we tested a new control participant, who took part in the two testing sessions, with a delay of circa one year between the first and the second experiment. Patients took part in the second experiment approximately 14.13 months (*SE* = 0.99) after the first; the interval between the first and second experiment in controls was 13.44 months (*SE* = 0.88).

The final sample consisted of sixteen patients (eight women, mean age at first testing: 39 years; range: 20–55) and sixteen age-, gender-, and education-matched control participants (eight women, mean age at first testing: 41 years, range: 19–58). We recruited and tested half of the patients in Berlin at the psychotherapy ambulance of the International Psychoanalytic University Berlin and half in Munich at the Department of Psychosomatic Medicine and Psychotherapy, München Klinik. Recruitment of patients happened in cooperation with a study on the efficacy of cognitive-behavioral and psychoanalytic therapy in panic disorder with comorbid personality disorder [31]. We recruited all control participants in Berlin through announcements on the web. The experiment took place at the International Psychoanalytic University Berlin and the Department of Psychosomatic Medicine and Psychotherapy, München Klinik.

According to the German version of the Structural Clinical Interview for DSM-4 [32], at the time of the first testing session, all the patients had a diagnosis of panic disorder with personality disorder comorbidity. None of the patients had eating, addictive, psychotic, or bipolar disorder. Eleven patients had panic disorder with agoraphobia, and half of the patients had a comorbid depressive disorder. The distribution of the personality disorders was ten avoidant, seven depressive, three dependent, three obsessive-compulsive; seven patients had more than one personality disorder. During the period between the first and second testing session, nine patients received psychoanalytic therapy, whereas seven received cognitive-behavioral therapy. Eight patients received the psychological treatment in combination with medications: seven patients took antidepressants, and one patient atypical antipsychotic medication because of its anxiolytic effect. Between the first and second testing session, one patient stopped the pharmacological therapy.

All participants had a normal or corrected-to-normal vision. According to the Edinburgh Handedness Inventory [33], participants were right-handed, except for one ambidextrous and two left-handed patients, and two left-handed controls. The ethics committee at the International Psychoanalytic University Berlin approved the study (protocol 2015–1), and participants gave their written informed consent before the beginning of each testing session. Participants received a monetary compensation of 20 € for their participation in the first testing session and 50 € for the second testing session.

### Procedure

This study had two testing sessions (T1 and T2), where participants performed the same tasks under identical experimental conditions. The experiment consisted of a response-choice task and a passive viewing task. The response-choice task started with a maximum of three practice blocks, to familiarize the participants with the stimuli and the relationship between performance and feedback, followed by twenty experimental blocks, divided into four runs. The passive viewing task was a sequence of pictures of faces or houses with a break after half of the stimuli. The response-choice task lasted approximately 45 minutes, whereas the passive viewing task lasted around 7 minutes. Both tasks were performed using Presentation^®^ software (Version 16.3, Neurobehavioral Systems, Inc., Berkeley, CA, www.neurobs.com).

Before the beginning of the experiment, participants filled the German versions [34–36] of the Penn State Worry Questionnaire [PSWQ; 37], the trait subscale of the State-Trait Anxiety Inventory form Y2 [STAI-t; 38], and the Beck Depression Inventory [BDI-II; 39].

#### Response-choice task

In the response-choice task, participants had to react to the identity of the central letter in a 3x3 array of the letters M, N, W, or H, arranged as a central letter (target) framed by eight identical letters (flankers). Participants had to press with the left or the right index finger one of two response buttons. A predetermined stimulus-button mapping assigned two letters (e.g., M and N) to one button and the other two letters (e.g., W and H) to the other button. The stimulus-button mapping was counterbalanced across participants. Stimulus arrays could appear above or below a central fixation cross. The two response buttons were placed vertically on the desk to match or mismatch the relative position of the stimulus array on screen, producing a cognitive conflict when the location of the response button was not congruent to the relative position of the stimulus array on screen [40]. The incongruency between the response elicited by the flankers and the response required by the target produced an additional cognitive conflict [41]. The parametric combination of four target letters, four flanker letters, and two array positions generated 32 different trials, performed once within each experimental block. Letters in the stimulus array (size of 0.32° x 0.32°, separated by gaps 0.05°) and the fixation cross (size: 0.32° x 0.32°) had a dark grey color (RGB: 78, 78, 78); the background had a light grey color (RGB: 128, 128, 128).

Within a trial, feedback appeared after each stimulus array. In half of the experimental blocks, feedback precisely reflected response quality (informative feedback). In this condition, a scrambled face appeared after errors or missing responses, while faces with a happy or neutral expression were feedback of correct fast and correct slow responses, respectively. In the other half of the experimental trials, scrambled faces were feedback of each response, irrespective of accuracy and speed (uninformative feedback). In the practice blocks, a red, green, or orange cross described performance accuracy and speed. The response speed of correct responses (fast or slow) was determined according to the median response time (RT) in the previous twenty-three correct trials. Feedback condition changed after five experimental blocks, resulting in the alternation between runs with informative feedback and runs with uninformative feedback. Instructions signaled a change of feedback condition before the beginning of a run. Each participant performed the same sequence of runs, with the same stimulus-response mappings, in both testing sessions.

Feedback stimuli were 170 pictures of neutral or happy faces from the stimuli set FACES [42], and 540 similar faces scrambled (10x10 pixel square size) with the Scramble plug-in (http://www.telegraphics.com.au/sw/product/Scramble) for Adobe Photoshop. All the stimuli were converted to greyscale and reshaped to fit a rectangular shape with rounded edges (size 6.81° x 4.52° of visual angle).

Each trial started with the presentation of the stimulus array, displayed on the screen for 250 ms, followed by a response period with a self-paced duration of maximum 1,250 ms. Feedback was presented for 1,000 ms after the end of the response period in trials with missing responses or 500 ms after the response. During the response period and the interval before feedback onset, the fixation cross was the only stimulus on screen. Trials were divided by 500-ms blank screens.

To invite a constant focus on the task, irrespective of the feedback condition, throughout the experiment, errors and slow responses were punished by the subtraction of 0.05 € and 0.02 €, respectively, from a starting bonus of 15.00 €. Participants were informed about the amount of money left in the bonus at the end of each run of five experimental blocks and received the bonus at the end of the experiment. Written feedback presented within the block invited the participant to be more accurate when accuracy in the last ten trials was below 50%. Moreover, at the end of each run, written feedback encouraged the participant to be faster or more accurate if the percentage of errors in the run distanced substantially from the ideal error rate of 10%.

#### Passive viewing task

In the passive viewing task, 150 pictures of houses and 150 pictures of faces with a neutral expression were presented randomly in sequence [stimuli for this task were taken from 43]. Stimuli were displayed for 1,000 ms, and they were separated one another by intervals of 500 ms blank screen. Instructions invited the participants to pay attention both to faces and houses. As an attentional check, for 1/7 of the pictures, the sequential presentation of stimuli stopped, and participants had to perform a 1-back recognition task, reporting whether the stimulus on screen was identical to (50% of the times) or different from (50% of the times) the stimulus presented immediately before.

### EEG recording and signal processing

The recording procedure at T1 and T2 was identical. The EEG was recorded by 28 Ag/AgCl electrodes mounted in an elastic cap (Easycap GmbH) with 2 additional Ag/AgCl electrodes applied directly on the skin over the left and the right mastoid (M1 and M2). According to the 10/20 System, the location of the electrodes in the cap corresponded to the positions Fp1/2, F7/8, F3/4, Fz, FC3/4, FCz, T7/8, C3/4, Cz, CPz, P7/8, P3/P4, Pz, PO7/8, PO9/10, O1/2, Oz. Additional 3 Ag/AgCl electrodes placed two on the outer canthi of the left and the right eye, and one below the right eye recorded the horizontal and vertical electrooculogram (EOG). During the recording, EEG and EOG signals were referenced to the left mastoid (M1), and the electrode AFz served as the ground.

EEG data were recorded with the software BrainVision Recorder (Brain Products GmbH, München, Germany) and analyzed with BrainVision Analyzer 2.1.2 (Brain Products GmbH, München, Germany). All signals were digitalized with a frequency of 500 Hz and filtered with a 0.05/70 Hz high/low pass filter. Electrodes’ impedance was smaller than 10 kΩ for all the electrodes. Offline, EEG and EOG signals were further filtered with a band-pass of 0.01 to 30 Hz and a slope of 48 dB/octave. Independent component analysis trained on calibration trials performed at the end of the experiment corrected the EEG signals from blinks, eye-movements, and pulse artefacts.

Response-locked and feedback-locked segments in the response-choice task and stimulus-locked segments in the passive viewing task started 200 ms before the marker of interest and lasted for 600 ms when response-locked or 1 second when feedback- or stimulus-locked. Based on visual inspection, segments with residual artifacts were discarded from the analyses. All segments were then re-referenced to the average activity of the mastoid electrodes (M1 and M2) and aligned to the 200 ms period preceding the 0 point of the segment.

Hajcak and Olvet [44] showed that six errors are the minimum number of trials for the calculation of reliable Ne/ERNs. Since seven patients and two controls made less than six errors in one of the feedback condition, trials with errors were considered together for the calculation of the average Ne/ERN, irrespective of feedback condition. An aggregation of incorrect trials between feedback conditions could not be performed for external signals because of the significant difference in the VPP evoked by scrambled faces in relation to the their information content [25]. Consequently, since 30 trials are necessary for reliable test-retest analysis of the VPP evoked by faces in a context of performance monitoring [45], we had to restrict the VPP analysis to feedback processing in trials with correct responses. The Ne/ERN was computed as the average activity at Fz between 0 and 100 ms of response-locked segments in trials with incorrect responses. The VPP was calculated as the peak-to-peak difference at Cz between the maximum negative amplitude between 100 and 160 ms and the maximum positive amplitude between 140 and 200 ms after feedback or stimulus onset.

In the present study, we tested a subsample of patients included in the study of Valt and collaborators [25]. Hence, as a precondition for the analysis of potential effects of treatment on abnormal ERPs in patients, we first checked whether the present sample of participants showed significant Ne/ERN and VPP differences at T1. To this end, since the direction of the effects were predicted, we investigated the ERPs evoked at T1 with one-tailed independent samples *t* tests.

For the determination of treatment-related changes of abnormal responses, ERPs evoked at T1 and T2 were considered in repeated measures ANOVAs. Testing Session (T1 vs. T2) and Group (patients vs. controls) were the only factors considered by the ANOVA performed on the Ne/ERN. Testing Session (T1 vs. T2), Performance (correct-fast vs. correct-slow), Feedback Condition (informative vs. uninformative feedback), and Group (patients vs. controls) were the factors of the ANOVA performed on the feedback-related VPP amplitude. The ANOVA on the stimulus-related VPPs considered the factors Testing Session (T1 vs. T2), Stimulus (faces vs. houses), and Group (patients vs. controls). The expected effect of treatment should result in a significant interaction between Group and Testing Session, with or without any further significant interaction with the other within-participant factors. Therefore, for clarity of the exposition, the results section focused on contrasts involving Group and Testing Session [for the exposition of main effects or interactions of Performance and Feedback Condition, see 25]. The significance level in the ANOVAs and the follow-up *t* tests was α = .05. All statistics were performed with IBM SPSS Statistics for Windows, Version 23 (Armonk, NY: IBM Corp).

## Results

### Clinical data

Table 1 reports the mean scores of the cumulative values in the three questionnaires. The analysis of the PSWQ showed a significant main effect of Group, *F*(1, 30) = 13.10, *p* = .001, *η*^*2*^_*p*_ = .304, but the main effect of Testing Session, *F* < 1, and the interaction between Testing Session and Group were both not significant, *F*(1, 30) = 1.22, *p* = .279. The absence of any significant change between T1 and T2 indicated that scores in the PSWQ were stable in both groups, meaning that treatment did not induce any significant reduction of worry in patients. The analysis of the trait subscale of the STAI showed a significant main effect of Group, *F*(1, 30) = 10.03, *p* = .004, *η*^*2*^_*p*_ = .250, a significant main effect of Testing Session, *F*(1, 30) = 4.36, *p* = .045, *η*^*2*^_*p*_ = .127, but no interaction between these two factors, *F*(1, 30) = 2.66, *p* = .113. These results revealed that participants reported overall lower anxiety symptoms in the second testing session but the numerically larger diminishment in patients was not significantly larger than the change in controls. In fact, patients presented higher anxiety in both testing sessions. Similarly, the analysis of the BDI showed a significant main effect of Group, *F*(1, 30) = 13.68, *p* = .001, *η*^*2*^_*p*_ = .313, a significant main effect of Testing Session, *F*(1, 30) = 23.64, *p* < .001, *η*^*2*^_*p*_ = .441, but the interaction between Group and Testing Session was short of significance, *F*(1, 30) = 3.82, *p* = .060. Noteworthy, in patients, the decrease in depressive symptoms was clinically relevant, even though, more than one year of psychotherapy, with or without medication, did not result in the complete remission of the depressive symptoms.

**Table 1:** Average scores in the questionnaires.

	Patients		Controls	
	T1	T2	T1	T2
**PSWQ**	47.06 (1.36)	44.00 (1.11)	40.88 (1.11)	40.50 (1.31)
**STAI-t**	56.50 (2.60)	54.00 (3.31)	40.38 (3.33)	40.59 (2.74)
**BDI**	21.94 (2.67)	14.34 (1.75)	6.84 (2.01)	4.50 (1.59)

PSWQ: Penn State Worry Questionnaire; STAI-t: trait subscale of State-Trait Anxiety Inventory; BDI: Beck Depression Inventory; T1: first testing session; T2: second testing session

### Behavioral results

#### Response-choice task

After the exclusion of missing or too early responses (RT < 250 ms), average accuracy and RTs (see Table 2) were calculated for the two experimental conditions (informative feedback and uninformative feedback) at T1 and T2. In the analysis of accuracy, Feedback Condition was significant as a main effect, *F*(1, 30) = 48.15, *p* < .001, *η*^*2*^_*p*_ = .616, revealing that responses were less accurate in the condition with informative feedback. Interestingly, the interaction between Group and Testing Session was also significant, *F*(1, 30) = 5.13, *p* = .031, *η*^*2*^_*p*_ = .146. In the absence of any other significant main effects or interactions, *F*s(1, 30) < 1.76, *p*s > .194, this result reflected a significant increase of accuracy at T2 in patients, *F*(1, 15) = 20.24, *p* < .001, *η*^*2*^_*p*_ = .574, but no difference in accuracy between T1 and T2 in controls, *F*(1, 15) < 1. In the analysis of RTs, besides the significant main effect of Feedback Condition, *F*(1, 30) = 20.37, *p* < .001, *η*^*2*^_*p*_ = .404, indicative of slower responses in the condition with uninformative feedback, no other main effect or interaction was significant, *F*s(1, 30) < 2.78, *p*s > .106, suggesting that patients and controls had similar response speed in both testing sessions.

**Table 2:** Behavioral results.

	Patients		Controls	
	T1	T2	T1	T2
**Response-choice task**				
**Accuracy**				
**• informative feedback**	88.43 (1.46)	91.94 (1.13)	88.59 (1.30)	87.18 (2.29)
**• uninformative feedback**	91.67 (1.11)	94.86 (0.97)	91.47 (0.95)	90.85 (1.65)
**RT**				
**• informative feedback**	677 (19)	676 (20)	707 (19)	691 (27)
**• uninformative feedback**	710 (20)	691 (19)	728 (19)	711 (26)
**Passive viewing task**				
**Accuracy**				
**• Faces**	90.31 (2.68)	97.19 (1.12)	91.25 (2.02)	93.44 (2.03)
**• Houses**	89.38 (2.02)	93.44 (1.69)	85.94 (2.51)	87.50 (2.77)

Accuracy is expressed in percentages of correct responses; RTs are expressed in ms

#### Passive viewing task

The recognition accuracy (see Table 2) in the 1-back recognition task was significantly higher for faces than houses, as indexed by the significant main effect of Stimulus, *F*(1,30) = 9.38, *p* = .005, *η*^*2*^_*p*_ = .238. Moreover, participants were more accurate at T2 compared to T1, *F*(1,30) = 5.49, *p* = .026, *η*^*2*^_*p*_ = .155. This performance improvement was independent of Group and Stimulus, as indicated by the absence of any significant interaction between these two factors and Testing Session, *F*s(1,30) < 1.59, *p*s > .217.

### ERP results

#### Response-choice task

At T1, the Ne/ERN analysis showed that errors evoked a significantly larger Ne/ERN in patients than in controls, *t*(30) = 1.72, *p* = .049. This result indicated that the subsample of participants taking part in both T1 and T2 was representative of the larger sample of participants described in Valt et al. [25].

Table 3 reports the mean amplitudes of the Ne/ERN in patients and controls across the two testing sessions. The ANOVA showed that errors at T1 and T2 evoked Ne/ERNs with similar amplitudes, as indexed by the not significant main effect of Testing Session, *F*(1, 30) = 2.87, *p* = .100, and the not significant main effect of Group, *F*(1, 30) = 1.04, *p* = .316. Moreover, the absence of a significant interaction between Group and Testing Session, *F*(1, 30) = 2.82, *p* = .104, indicated that treatment did not determine any significant change in patients.

**Table 3:** Electrophysiological results of internal signal processing (Ne/ERN amplitude) and external signal processing (VPP peak-to-peak value) in the response-choice task and the passive viewing task.

	Patients		Controls	
	T1	T2	T1	T2
**Response-choice task**				
**Ne/ERN**	-3.57 (0.44)	-3.57 (0.62)	-2.09 (0.74)	-3.58 (0.55)
**VPP (feedback)**				
**• informative fast**	19.26 (1.19)	17.22 (1.20)	14.42 (1.20)	15.98 (1.47)
**• informative slow**	19.38 (1.31)	17.62 (1.32)	15.07 (1.24)	15.28 (1.24)
**• uninformative fast**	12.20 (1.15)	11.24 (1.04)	9.08 (1.16)	10.18 (1.24)
**• uninformative slow**	12.13 (1.08)	10.58 (1.03)	8.09 (1.26)	8.75 (1.19)
**Passive viewing task**				
**VPP (stimulus)**				
**• Faces**	17.51 (1.31)	14.73 (1.25)	12.53 (1.22)	13.13 (1.40)
**• Houses**	11.03 (1.03)	9.01 (0.97)	8.22 (0.96)	8.53 (1.05)

Mean values (standard errors) are expressed in μV

Reverting to the processing of external signals, the analysis restricted to the VPP at T1 showed a significant main effect of Group, *t*(30) = 2.63, *p* = .007. Hence, before the beginning of treatment, patients with panic disorder presented abnormal VPP amplitudes evoked by external signals.

**Fig 1:**
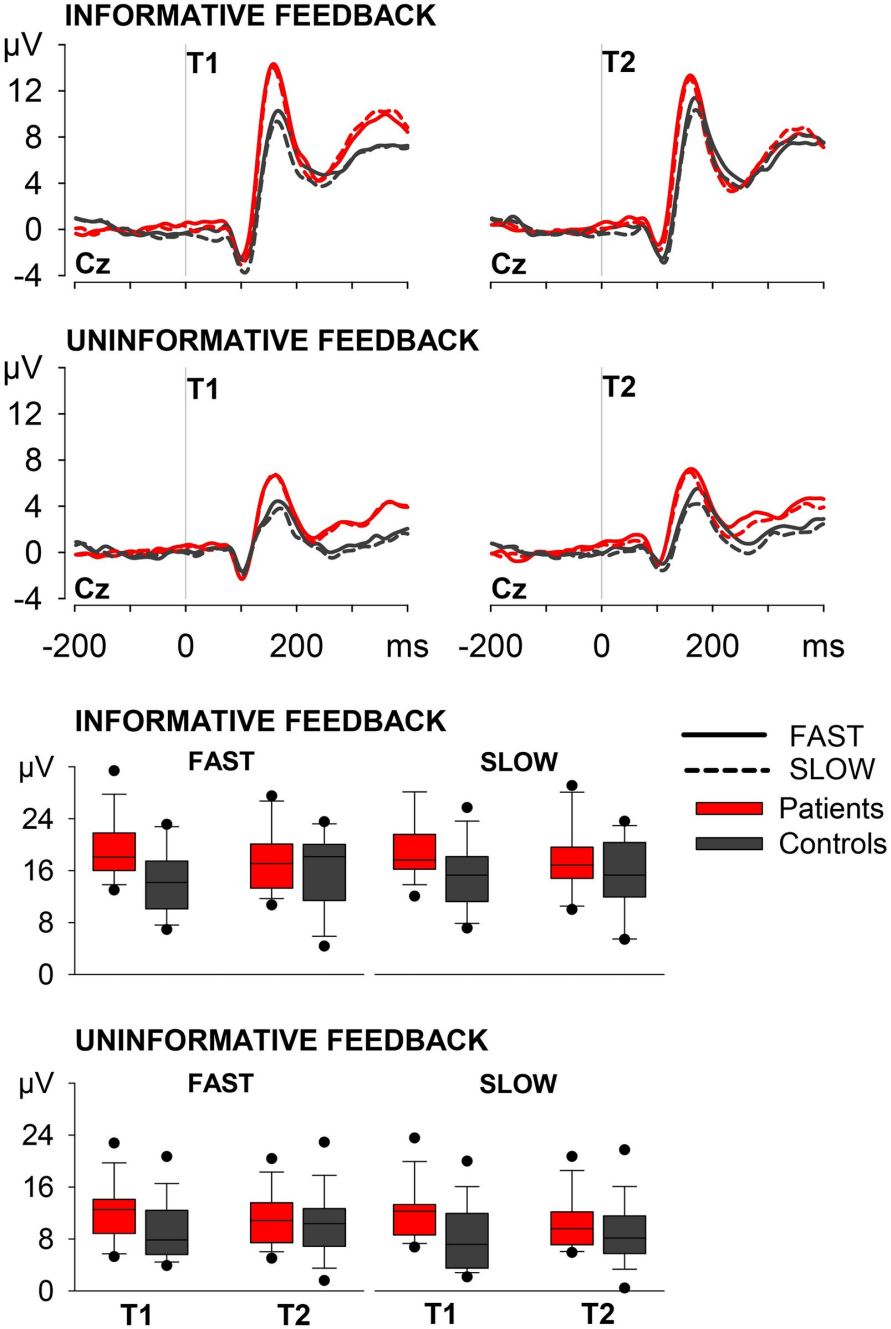
Feedback-locked ERPs. Grand average ERPs at Cz for trials with correct-fast and correct-slow responses in patients and controls. Box plots of the median VPP in the two groups at T1 and T2, separately for the two feedback conditions.

Table 3 reports the peak-to-peak values of the VPP in the different experimental conditions, whereas Fig 1 depicts the grand average ERPs used for the calculation of the VPPs. Whether treatment determined a significant reduction of the observed abnormal VPP amplitudes in patients was the focus of the contrasts between Group and Testing Session in the ANOVA on the VPPs evoked at T1 and T2. Testing Session and Group were both not significant when considered as main effects, *F* < 1, and *F*(1, 30) = 3.77, *p* = .062, respectively, but they showed a significant interaction, *F*(1, 30) = 5.02, *p* = .033, *η*^*2*^_*p*_ = .143. In agreement with the expected reduction of VPP amplitudes after treatment, patients presented significantly smaller VPPs at T2 compared to T1, *F*(1, 15) = 7.04, *p* = .018, *η*^*2*^_*p*_ = .319, whereas no significant difference between testing sessions was observed in controls, *F*(1, 15) < 1. Interestingly, the interaction between Group and Testing Session was further qualified by the interaction with the other two within-participant factors, *F*(1, 30) = 4.30, *p* = .047, *η*^*2*^_*p*_ = .125 (see Fig 1), suggesting that the treatment-related changes of VPP amplitudes in patients were affected by feedback quality. In the condition with informative feedback, the reduction of VPP amplitude between T1 and T2 was significant both in trials with correct-fast responses, *F*(1, 15) = 9.66, *p* = .007, *η*^*2*^_*p*_ = .392, and in trials with correct-slow responses, *F*(1, 15) = 4.84, *p* = .044, *η*^*2*^_*p*_ = .244. However, in the condition with uninformative feedback, the reduction of VPP amplitude between T1 and T2 was not significant in trials with correct-fast responses, *F*(1, 15) = 1.93, *p* = .186, but significant in trials with correct-slow responses, *F*(1, 15) = 9.23, *p* = .008, *η*^*2*^_*p*_ = .381. In controls, instead, none of the VPPs evoked by feedback showed any significant change, *F*s(1, 15) < 1.37, *p*s > .189.

The observed VPP reductions between T1 and T2 in patients determined a normalization of the originally abnormal response evoked by feedback signals in patients, as indicated by the not significant main effect of Group at T2, *t*(30) < 1.

#### Passive viewing task

Before the beginning of treatment, patients with panic disorder showed enhanced VPP, as indicated by the significant effect of Group in the analysis of the VPP evoked by faces and houses at T1, *t*(30) = 2.66, *p* = .012. In conformity to the results reported in Valt et al. [25], before the beginning of therapy, the present sample of patients showed an enhanced VPP in the processing of stimuli for the passive viewing task.

**Fig 2:**
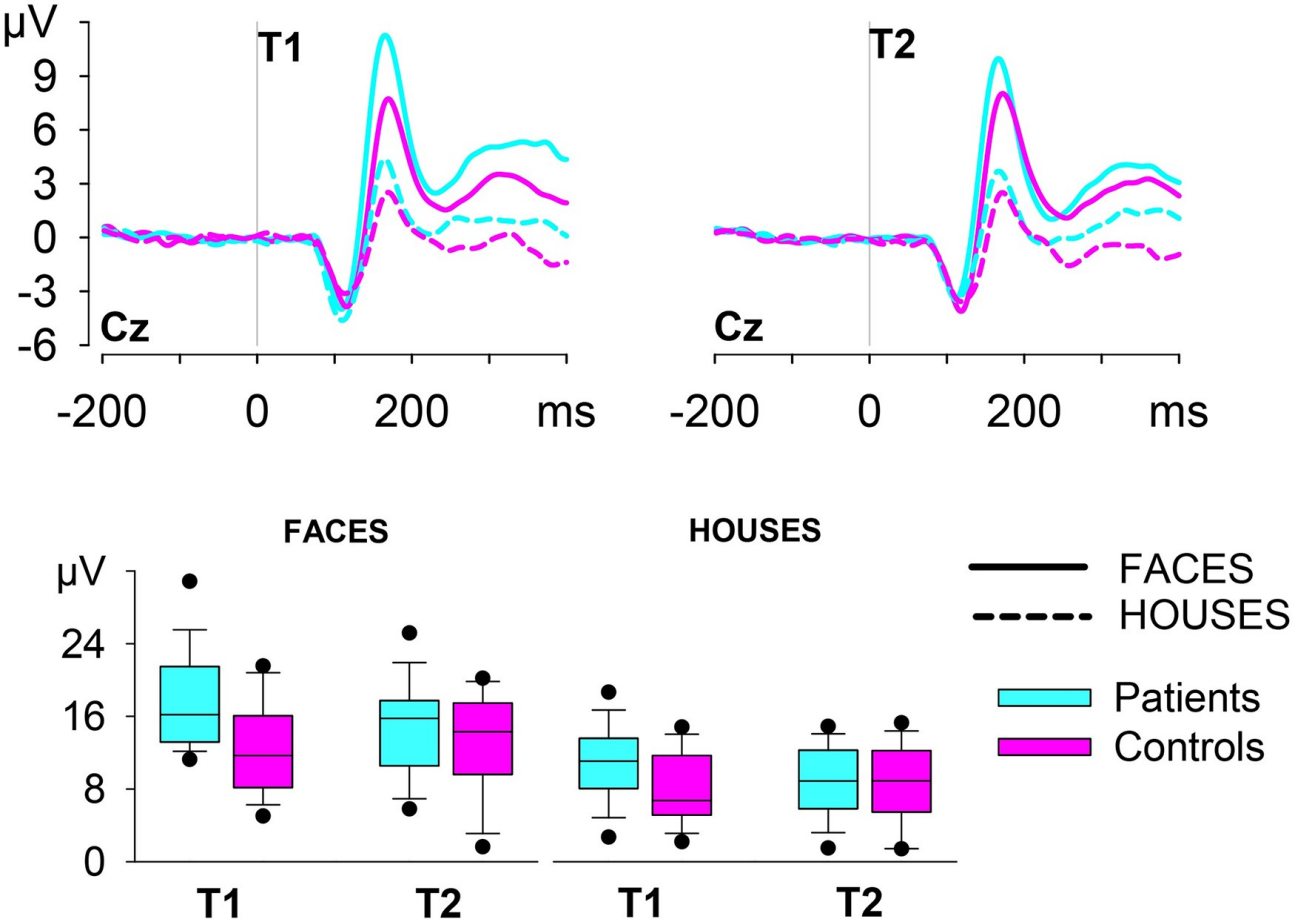
Stimulus-locked ERPs. Grand average ERPs at Cz for the processing of stimuli in patients and controls. Box plots of the median VPP in the two groups at T1 and T2, separately for faces and houses.

The VPP amplitudes at T2 were then incorporated in the analysis to investigate potential reductions of the abnormal VPP activity in patients (see Fig 2 and Table 3). Testing Session and Group were both short of significance when considered as main effects, *F*(1, 30) = 3.48, *p* = .072, and *F*(1, 30) = 3.05, *p* = .091, respectively, but they presented a significant interaction, *F*(1,30) = 7.46, *p* = .010, *η*^*2*^_*p*_ = .199 (see Fig 2). In patients, the VPPs evoked by stimuli in the passive viewing task were smaller at T2 than at T1, *F*(1, 15) = 18.49, *p* = .001, *η*^*2*^_*p*_ = .552, but they did not present any significant change across testing sessions in controls, *F*(1, 15) < 1. Since none of the other interactions was significant, *F*s(1,30) < 1.91, *p*s > .177, these results indicated that treatment determined a significant change towards normalization of the VPP amplitude evoked by stimuli in the passive viewing task, irrespective of their visual characteristics (faces or houses).

Similarly to the results in feedback processing, the missing main effect of Group at T2, *t*(30) < 1, indicated that treatment determined a normalization of the processing of external signals.

## Discussion

The present experiment investigated the effect of psychotherapy on the electrophysiological response evoked by external and internal signals. Contrary to previous performance monitoring studies that analyzed exclusively the processing of errors based on internal signals, we focused on the processing of external signals, indexed by the VPP, and extended the investigation to the passive viewing of faces or houses. This investigation should clarify whether treatment can affect early information processing. The Ne/ERN results did not show any effect of treatment on the processing of errors based on internal signals. However, in patients, treatment determined significant changes towards normalization of the abnormal VPP evoked by feedback in the response-choice task and by visual material in the passive viewing task. Hence, the present results show that treatment determined a normalization of abnormal processing of external signals, irrespective of their meaning (informative feedback or uninformative feedback) and semantic category (pictures of faces or houses).

In the present study, 16 patients with a diagnosis of panic disorder with comorbid personality disorder and their respective 16 controls performed a response-choice task and a passive viewing task in two testing sessions. The interval between the first and the second testing sessions was circa 14 months, during which patients received psychotherapy. During this period, half of the patients took antidepressants, but there was only a minor change in medication between the two testing sessions (one patient stopped taking medications). The analysis of the data of the first testing session indicated that the subsample of participants tested for the present investigation was representative of the larger sample tested in Valt et al. [25]. In the present study, before the beginning of treatment, patients showed enhanced Ne/ERN evoked by errors and augmented VPP evoked by visual material in both the response-choice task and the passive viewing task.

In patients, the enhanced VPPs evoked by visual stimuli suggested heightened processing of external signals, irrespective of their semantic category and meaning. The comparison between the first and second testing session revealed a significant reduction of the VPP amplitude in patients, while controls did not show any significant change between testing sessions. This treatment-related change in VPP amplitude in patients revealed normalization of the initially abnormal processing of external signals, as indicated by the absence of any significant difference between groups of the VPP amplitudes recorded in the second testing session. Therefore, the present results extend previous fMRI and EEG observations of treatment-related modulations of visual stimuli [2–4]. Buchheim and collaborators [4] observed treatment-related changes in the late positive potential evoked by emotional material between 600 and 1000 ms after stimulus onset. Here, we observed the effect of treatment on the VPP evoked at around 200 ms after the stimulus. This result indicated that psychotherapy can affect early stages of information processing of visual material. Moreover, previous studies showed effects of treatment on the processing of emotional material [2–4], such as attachment projective pictures. Here, we recorded significant changes not only for emotional faces, used as informative feedback of performance, but also for scrambled faces presented as uninformative feedback of correct-slow responses and for pictures of houses in the passive viewing task. Therefore, the present results indicate that psychotherapy might have an effect on the processing of visual material, irrespective of its emotional content. However, future research is required to qualify the relevance of face processing in detecting treatment-related changes. The results showed that, when the external signal was a neutral or a happy face, the VPP reduction was significant in both trials with correct-fast and correct-slow responses, whereas, when the external signal was always a scrambled face, a significant VPP reduction occurred only in trials with correct-slow responses. This result could indicate that the processing of unmodified faces is more sensitive to the effect of treatment. However, in the present experiment, the confound of feedback informativeness and face processing does not allow the precise characterization of this possibility.

Studies of attention in face processing have shown that the allocation of attention enhances the amplitude of the N170 [46]. In the context of performance monitoring, directing participants’ focus to feedback processing determines an enhancement of the N170 [47]. Hence, the present results might indicate a better allocation of attentional resources to external signals, with a consequential reduction of hypervigilance in panic disorder. Importantly, this result does not reflect a disengagement from the task, resulting in the subtraction of attentional resources irrespective of treatment. In fact, in the passive viewing task, where attention on visual material was relevant for the recognition task, patients were overall more accurate in the second than the first testing session. Moreover, the significantly better performance of patients compared to controls in the response-choice task indicates that patients were more engaged in this task as well. Therefore, the present results could indicate that treatment determined a correction of the pathological hypervigilance on external signals in patients, leading to an improvement of performance. Importantly, the questionnaires showed that, in the second testing session, patients still presented high depressive and anxiety symptoms. In fact, one year after the beginning of treatment, all the patients were still in therapy, meaning that remission of the panic disorder, along with the depressive symptoms, was not complete. Future studies should replicate the present results after the conclusion of therapy, to check whether treatment can lead to a stable normalization of the abnormal VPP amplitudes in patients suffering from panic disorder with comorbid personality disorder and whether this electrophysiological parameter can be predictive of a potential return of symptoms.

Contrary to the observation of significant effects of treatment on the processing of external signals, the analysis of the Ne/ERN failed to highlight any treatment-dependent normalization of internal processing of errors in patients. This result conforms to previous studies of internal signal processing after treatment [7–11]. Hence, the present Ne/ERN results are in line with previous observations that treatment does not reduce the enhanced processing of errors in patients, in satisfaction of the state-independency criterion of endophenotypes [20]. However, one limitation of the present experiment does not permit the advancement of solid conclusions on whether treatment affected the amplitude of the Ne/ERN in patients. The significant increment of accuracy in patients at T2 could have changed the significance of errors. Holroyd and Coles (19) showed that the Ne/ERN is related to the number of errors, with augmented amplitudes when errors are rare, and therefore more salient. In the present experiment, an increase of the Ne/ERN amplitude linked to the higher relevance of rare errors in T2 might have masked significant reductions of the abnormal Ne/ERN resulting from treatment.

In the context of performance monitoring, this result is the first evidence of treatment-related normalization of abnormal brain responses in patients suffering from an internalizing psychological disorder. Previous studies of performance monitoring failed to observe any significant change of the Ne/ERN in patients after treatment [7–11], but they neglected potential changes in other abnormal responses, like the VPP or the FRN evoked by feedback [12, 21, 25]. Here, instead, we focused on the processing of external signals, and the observed treatment-related ERP changes indicate that, compared to the Ne/ERN, the VPP might be an electrophysiological response more suitable for studying beneficial effects of treatment on abnormal brain functioning in internalizing psychopathologies. Since the VPP is an ERP evoked by the processing of visual material irrespective of the context, in future experiments, there is no need to employ a performance monitoring paradigm with feedback to explore the VPP. A simple experimental design, such as the passive viewing task performed in the present study, is sufficient for adequate investigation of treatment-related changes in early information processing.

The present results represent a starting point for future investigations on the effects of treatment on the normalization of abnormal brain functions. However, future experiments, with larger samples of patients, are required to determine whether psychoanalytic therapy and cognitive-behavioral therapy lead to comparable changes in brain functioning. Moreover, the relevance of pharmacotherapy is an additional factor to be addressed. Further research is also required to understand whether an abnormal VPP evoked by external signals is specific to panic disorder or whether it is an aspect common to all psychological disorders characterized by internalization. It might be seen as a limitation that, in our clinical sample, more than half of the patients had a comorbid depressive disorder. However, psychological comorbidities are frequently observed [25], especially for generalized anxiety disorder, panic disorder, and social phobia (80–90%). Moreover, the relevance of comorbid personality disorder for the observed abnormal ERPs is a factor that calls for close scrutiny.

In conclusion, the present experiment is the first evidence of treatment-related changes towards normalization of abnormal brain activities in early information processing of visual stimuli. According to this result, psychotherapy seems to be effective both for the patients’ psychological well-being and for the normalization of abnormal brain functions.
